# First-Principles Simulation of Anharmonic and Anisotropic Vibrations of Glycinate on Copper

**DOI:** 10.1021/acsomega.5c00210

**Published:** 2025-02-13

**Authors:** Alexander D. Ievins, Marco Sacchi, Stephen J. Jenkins

**Affiliations:** † Yusuf Hamied Department of Chemistry, University of Cambridge, Lensfield Road, Cambridge CB2 1EW, U.K.; ‡ School of Chemistry and Chemical Engineering, University of Surrey, Guildford GU2 7XH, U.K.

## Abstract

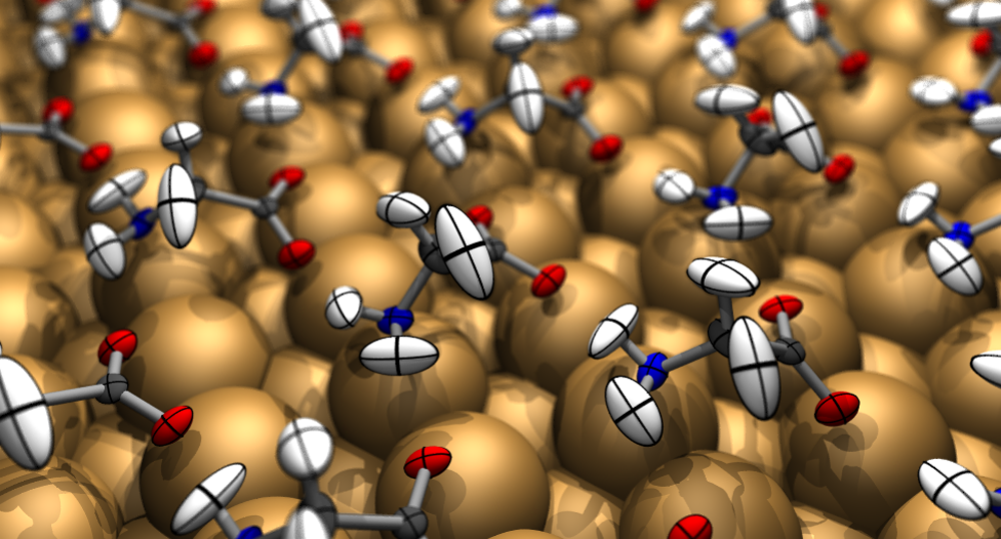

Molecular vibrations within a hydrogen-bonded network are expected to be significantly anharmonic and hence poorly described by conventional normal-mode analysis. Moreover, the rather flat potential energy landscapes experienced in such cases imply sampling of several local-energy minima, casting further doubt upon the standard methodology. Both difficulties may be overcome through first-principles molecular dynamics, used here to obtain vibrational spectra and thermal ellipsoids for glycinate adsorbed on copper. Vibrational anisotropy and signatures of hydrogen bonding are highlighted and discussed.

## Introduction

Density functional theory (DFT) has, for decades, been the method of choice for first-principles calculations relating to the adsorption and reaction of molecules at solid surfaces. Structural, thermodynamic, and kinetic properties are often obtained to a pleasing degree of accuracy, albeit subject to occasional instances that caution against complacency. For reasons of computational economy, however, the vast majority of such calculations have typically been executed in what may be termed static (geometry optimization) or quasi-static (transition-state search) modes of operation. DFT-based ab initio molecular dynamics (AIMD) calculations remain, for surface studies, comparatively rare.

That said, it has long been clear that dynamic calculations offer notable advantages over static and quasi-static approaches. Reaction studies, for example, need not be confined to thermal processes (with kinetics derived from transition-state theory) but can include instances where molecules start or end in highly nonequilibrium states of nuclear motion, as may occur during scattering, adsorption, desorption, or diffusion.^1−24^ Such processes typically sample highly anharmonic regions within the potential energy landscape, which AIMD can reproduce on-the-fly as necessary. In a similar vein, the present work employs AIMD to obtain information about highly anharmonic nonreactive surface vibrational states, including the derivation of thermal ellipsoids and vibrational spectra, for a system in which traditional normal-mode analysis is rendered suspect by hopping between multiple local-energy minima. In such cases, we argue that dynamic calculations are highly desirable if the goal is to make better contact with structural techniques (e.g., low-energy electron diffraction, LEED) or spectroscopic techniques (e.g., reflection absorption infrared spectroscopy, RAIRS).

The molecule chosen to exemplify this approach is the amino acid glycine, which is known to adsorb on Cu{110} in its deprotonated nominally anionic form (i.e., as glycinate) with (3 × 2) periodicity at a surface coverage of 1/3 ML.^25−32^ This system provides a stringent test in that it displays a complex vibrational spectrum combining low-frequency torsions, a mid-frequency fingerprint region, and high-frequency stretch modes likely to be affected by hydrogen bonding. Moreover, it has been thoroughly studied by both experimental and computational means. In a crucial early work, Barlow et al.^25^ interpreted their RAIRS data as indicating three-point binding via the molecule’s two oxygen atoms and its single nitrogen atom, and subsequent DFT calculations^26−31^ concur that this assignment is very likely correct. Each molecule stretches from one of the close-packed rows on the surface to a neighboring row, occupying a locally chiral “footprint” taking the form of a right-angled triangle of copper atoms; its backbone may either be kinked to one side or another or remain essentially linear when viewed along the surface normal. Multiple different local configurations are therefore possible, depending upon the backbone conformations adopted by adjacent molecules, and many of these are found to have extremely similar energies within the accuracy of DFT.^29^ Not only is the energetic cost of perturbing the molecular backbone very small but the network of intramolecular hydrogen bonds is also rather insensitive to such distortions. As each molecule twists, some of its hydrogen bonds may weaken, but others invariably strengthen in compensation.

## Methodology

Our present DFT calculations were performed using the CASTEP code (Version 18.1)^33^ employing similar convergence parameters to a previous work.^29^ Precise details are provided in the Supporting Information (SI). The supercell dimensions were chosen consistent with a (3 × 2) surface unit cell, containing either one molecule (1/6 ML) or two independent molecules (1/3 ML). The AIMD simulations employed a five-link Nosé–Hoover thermostat chain (set to maintain 500 K) and a time step of 0.25 fs. Since the initial motion in each simulation may be unduly influenced by the choice of starting geometry, data from a 1 ps equilibration period was omitted from the subsequent analysis. Our calculated trajectory for each phase then extended for 9 ps after the equilibration period, during which substantial oscillatory deformation of the adsorbed molecules was observed. The extent of this deformation was sufficient to sample regions of configuration space reminiscent of several different local-energy minima previously reported for this system, confirming that normal-mode analysis based on a single harmonic potential-energy well would be incapable of capturing the entire vibrational spectrum. Instead, we extract structural and spectroscopic information directly from our AIMD simulations, using a suite of in-house computer codes described in the SI and available for download (https://doi.org/10.17863/CAM.115737).

## Results and Discussion

We begin our analysis by investigating the mean atomic positions, which are straightforwardly obtained by averaging Cartesian coordinates over the post-equilibration data set. The resulting mean structure for the 1/3 ML phase displays an approximate glide symmetry, relative to which we compute root-mean-square (rms) deviations from perfection of 0.05 Å across all adsorbate atoms, 0.03 Å for the top-layer copper atoms, and just 0.01 Å for the second substrate layer. Since we have not actively imposed any symmetry constraints during the calculations, we take this as evidence that our simulations have run for sufficient time to adequately sample the configuration space of the system (see the SI for further discussion on this point). Note that the instantaneous geometry *never* displays such a clear glide symmetry (with rms deviations from perfection typically at least an order of magnitude larger than those reported above for the mean structure) but that experimental LEED data *does*.^25^ It is thus the time-averaged atomic positions that provide the closest match to LEED, which itself averages spatially over hundreds of unit cells. Photoelectron diffraction (PhD), on the other hand, derives from emission localized at individual molecules,^34,35^ but each one may be caught at a different point in its oscillation when that emission occurs; once again, we expect the time-averaged atomic positions to give the best structural comparison to such data.

Beyond simply revealing the mean atomic positions, however, the data also allow us to address vibrational motion around those positions. We do this by constructing a thermal ellipsoid for each atom according to the method described by Kronenburg.^36^ Defining *u*_*i*_ (*i* = *x*, *y*, *z*) as the instantaneous Cartesian displacement of an atom from its mean position, we construct the atomic displacement matrix **U** = ⟨*u*_*i*_*u*_*j*_⟩ and obtain its eigenvalues, , and normalized eigenvectors, **w**_*n*_ (*n* = 1, 2, 3). The semiaxes of the desired thermal ellipsoid for this atom then lie in the directions of the eigenvectors, with lengths proportional to the square root of the corresponding eigenvalue. For reference, the scaling factor recommended by Kronenburg^36^ (and adopted here) yields ellipsoids that enclose slightly more than half the total probability of finding the corresponding atom at any given instant.

**Figure 1:**
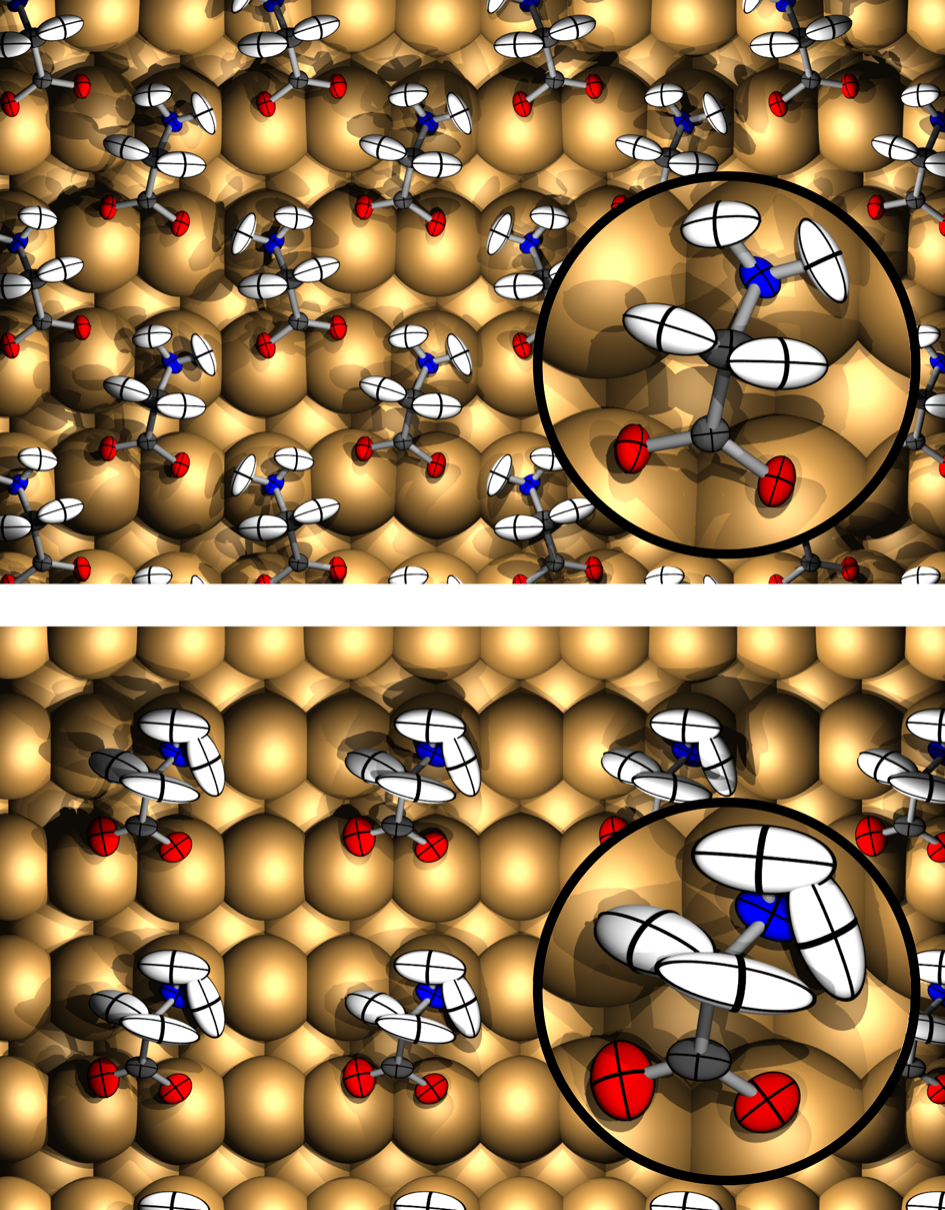
Thermal ellipsoids calculated at 500 K for glycinate on Cu{110} at 1/3 ML (upper panel) and 1/6 ML (lower panel). White, gray, blue, and red ellipsoids indicate H, C, N, and O atoms, respectively, while Cu atoms are represented as just-larger-than-touching spheres (not thermal ellipsoids).

Figure 1 (upper panel) shows the result of such an analysis for the 1/3 ML phase of glycinate on Cu{110}, in which we have imposed perfect glide symmetry on the displacement matrices prior to diagonalization (see the SI for nonsymmetrized results). A number of interesting features are apparent. First, the ellipsoids are highly anisotropic, with those of the hydrogen atoms in particular showing considerable elongation in directions consistent with predominantly scissor- and rocking-mode vibrations. Second, the ellipsoid orientations are nearly mirror-symmetric with respect to the backbone of each molecule (see inset). In contrast, the ellipsoids in the 1/6 ML phase are markedly asymmetric in their orientations, reflecting a similar asymmetry in the mean atomic positions (lower panel of Figure 1). Notably, the ellipsoids of all of the atoms (but especially the hydrogen atoms) are also considerably larger relative to the higher-coverage phase, indicating a much greater range of motion. More subtly, the motion of the oxygen atoms in the direction parallel to the surface’s close-packed rows is disproportionately reduced at high coverage, changing their ellipsoids from approximate oblate spheroids (with the shortest axis nearly normal to the surface) to near-perfect prolate spheroids (with the longest axis directed mainly across the close-packed rows). All of these changes reflect an effective stiffening of the molecular backbone in the high-coverage case, presumably linked to the constraint imposed by incorporation into the hydrogen-bonded network.

Taken together, the time-averaged positions and thermal ellipsoids obtained from AIMD represent a significant improvement upon static calculations in the context of comparison with diffraction experiments on such flexible systems. In particular, the indication of considerable anisotropy in thermal motion could usefully inform the choice of Debye–Waller factors in diffraction analysis (usually assumed to be approximately isotropic). It is to be hoped that inclusion of anisotropic thermal effects in future diffraction analyses will provide a pertinent test of the present computational results.

**Figure 2:**
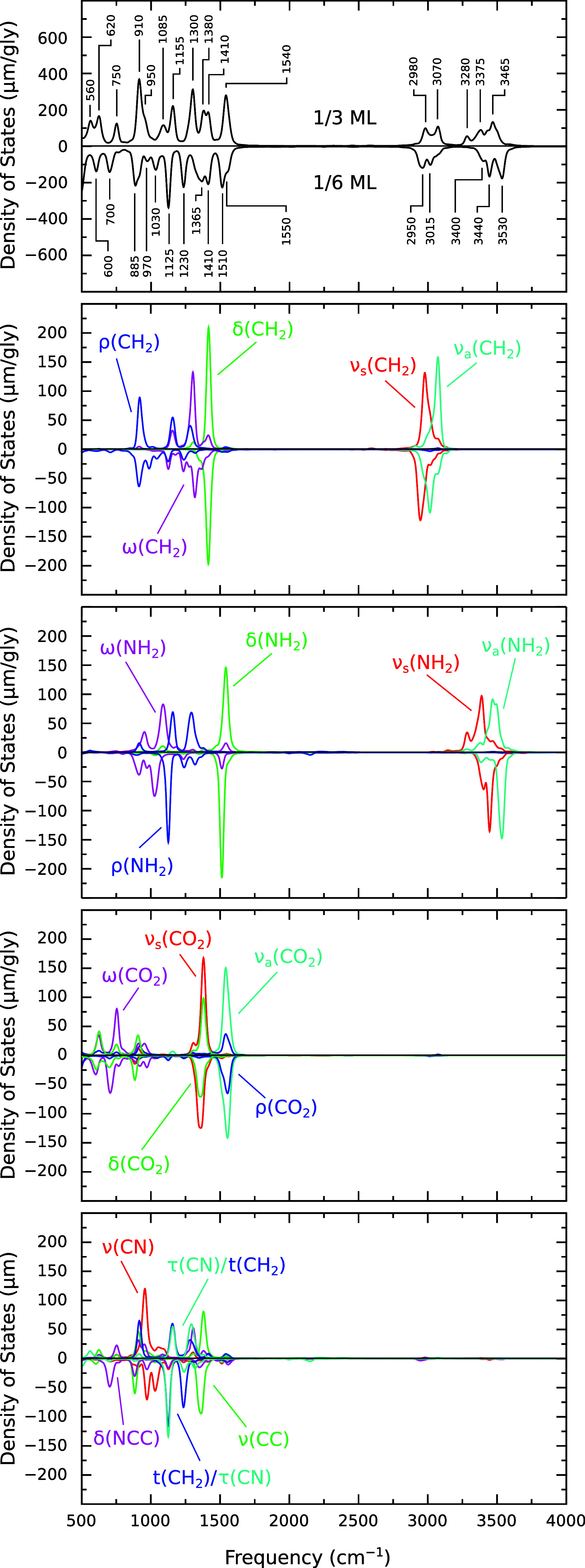
Vibrational DOS for adsorbed glycinate at 1/3 ML and 1/6 ML coverages, normalized on a per-adsorbate basis.

In addition to connecting with structural inferences drawn from diffraction experiments, however, AIMD calculations also have much to contribute toward the interpretation of spectroscopic data, particularly where anharmonicity and/or the sampling of multiple local minima may be important. To proceed, we calculate the power spectral density of mass-weighted velocity presented in the upper panel of Figure 2 for both 1/3 ML and 1/6 ML coverages. Note that the Wiener–Khintchine theorem^37^ asserts this to be entirely equivalent to evaluating the Fourier transform of the mass-weighted velocity autocorrelation function (see the SI). At equilibrium, the equipartition theorem implies that each mode will contribute equal kinetic energy to these distributions, in which case they may sensibly be interpreted as vibrational densities of states (DOSs).^38^ In the broadest of terms, the region below around 250 cm^–1^ (not shown in the figure, but see the SI) is dominated by substrate phonon modes, giving way to frustrated rotations and translations before entering into the fingerprint region spanning the 500–1550 cm^–1^ range (and comprising multiple overlapping intramolecular modes). On the other side of an extended flat region, peaks within the 2850–3200 cm^–1^ range correspond more-or-less exclusively to CH_2_ stretch modes, while those within the 3200–3650 cm^–1^ range correspond similarly to NH_2_ stretch modes. The areas under various sections of the power spectrum curves correspond quite well with the number of states expected based on comparison with the gas-phase glycinate anion, indicating again that satisfactory equilibration and equipartition may reasonably be inferred (see the SI).

In order to gain further insight, we have also taken a slightly different approach, converting each surface trajectory from the original Cartesian coordinate system into a system of generalized local-mode coordinates adapted from those given by Shimanouchi^39^ and by Vijay and Sathyanarayana^40^ (see the SI). Having done this, we next evaluate the power spectral densities obtained from the corresponding local-mode velocities, each separately normalized to unity. The advantage of this approach is that it becomes rather straightforward to analyze the origin of individual peaks within the overall spectrum. Specifically, we can easily separate out contributions corresponding to individual local-mode coordinates that in turn may readily be interpreted as corresponding to symmetric and antisymmetric stretch modes, ν_s_ and ν_a_, for NH_2_, CH_2_, and CO_2_ (six in total); stretch modes, ν, for CN and CC bonds (two in total); torsional modes, τ, for CN and CC bonds (two in total); scissor, wag, and rocking modes, δ, ω and ρ, for NH_2_, CH_2_, and CO_2_ (nine in total); a twisting mode, *t*, for CH_2_ (one in total); and an additional scissor mode, δ, for NCC (one in total). These descriptors match the traditional designations used to describe group vibrations in the analysis of experimental spectra and are plotted within the lower panels of Figure 2 (averaged over two molecules per cell in the higher-coverage case). By comparing against the upper panel, we are readily able to assign local-mode character to the peaks of the full simulated spectra (see Table 1).

**Table 1:** Assignment of Peaks (frequencies quoted to the nearest 5 cm^–1^) in the Calculated Vibrational DOS for Glycinate on Cu{110}^a^

1/6 ML	1/3 ML	assignment
3530	3465	ν_a_(NH_2_)
3440	3375	ν_s_(NH_2_)
3400	3280	ν_s_(NH_2_)
3015	3070	ν_a_(CH_2_)
2950	2980	ν_s_(CH_2_)
1550	1540	ν_a_(CO_2_), ρ(CO_2_)
1510	1540	δ(NH_2_)
1410	1410	δ(CH_2_)
1365	1380	ν_s_(CO_2_), δ(CO_2_), ν(CC)
	1300	ω(CH_2_), ρ(NH_2_), τ(CN), δ(NCC)
1230		*t*(CH_2_), ω(CH_2_)
1125	1155	ρ(NH_2_), τ(CN), *t*(CH_2_), ρ(CH_2_), ω(CH_2_)
1030	1085	ω(NH_2_), ν(CN)
970	950	ν(CN)
	910	ρ(CH_2_), *t*(CH_2_), τ(CC), δ(NCC)
910		ρ(CH_2_), ω(NH_2_)
885		ν(CC), δ(CO_2_)
700	750	ω(CO_2_), δ(NCC)
600	620	δ(CO_2_), ω(CO_2_)
500	560	τ(CN), τ(CC)

a Where multiple local-mode assignments are given for a single peak, these are listed in descending order of their contribution.

In analyzing their RAIRS data for this system, Barlow et al.^25^ invoked comparison with reference systems to infer assignments for their observed spectral peaks. The assignments and peak positions obtained in the present work for a coverage of 1/3 ML are in gratifying agreement with the interpretation of that experimental study at the same coverage, barring a small number of relatively minor differences (Table 2). Experimental peaks at 2906 and 2860 cm^–1^ were previously assigned to antisymmetric and symmetric CH_2_ stretches, and we concur with the same assignments for our peaks at 3070 and 2980 cm^–1^. The conspicuous overestimation of these two frequencies stands in contrast to the slight underestimation of most other frequencies that can sensibly be compared with the experiment. For instance, a peak observed at 1417 cm^–1^ was originally assigned to the symmetric CO_2_ stretch and arguably corresponds most closely to a computed peak at 1380 cm^–1^ that involves significant motion of this type (albeit mixed with CO_2_ scissor and CC stretch contributions). A separate computed peak at 1410 cm^–1^ matches even more closely in frequency but has only CH_2_ scissor character, however, leaving the correct correspondence here a little ambiguous. On the other hand, the experimental peak reported at 1332 cm^–1^ and assigned originally to the CH_2_ wag mode corresponds well with a calculated peak at 1300 cm^–1^ that we indeed find to have primarily the same character, albeit combined with elements of NH_2_ rock, torsion around the CN bond, and NCC scissor motion.

**Table 2:** Reconciliation between Observed^25^ and Calculated Peaks (frequencies in cm^–1^) in the Vibrational DOS of 1/3 ML Glycinate on Cu{110}^a^

RAIRS	MD	assignment
2906	3070	ν_a_(CH_2_)
2860	2980	ν_s_(CH_2_)
1417	1410	δ(CH_2_) instead of ν_s_(CO_2_)
1417	1380	ν_s_(CO_2_) but also δ(CO_2_), ν(CC)
1332	1300	ω(CH_2_) but also ρ(NH_2_), τ(CN), δ(NCC)
1084	1085	ω(NH_2_), ν(CN)
969	950	ν(CN) instead of ν(CC)
945	910	ρ(CH_2_) but also *t*(CH_2_), τ(CC), δ(NCC)

a Identifications are in agreement with the original assignments except where explicitly noted. Two possible correspondences are provided in one ambiguous case, discussed further in the main text.

Continuing the assignment to lower frequencies, the peak found at 1084 cm^–1^ in the RAIRS experiment is reproduced by our calculations at 1085 cm^–1^ and assigned, both then and now, to a combination of NH_2_ wagging motion and CN stretch. Similarly, we agree that the observed peak at 945 cm^–1^ (found here at 910 cm^–1^) is dominated by CH_2_ rocking motion (albeit with some CH_2_ twist, CC torsion, and NCC scissor). The only real discrepancy, then, relates to the peak observed in the experiment at 969 cm^–1^ and attributed to the CC stretch. We find only one otherwise unattributed peak in this frequency range, at 950 cm^–1^, but instead assign it predominantly to the CN stretch.

Note that calculated peaks at 3465, 3375, 3280, 1540, and 1155 cm^–1^ ought not to be expected in the experimental spectra since all predominantly involve oscillations that imply only very small dynamic dipole moments in the surface-normal direction. The peaks predicted at 750, 620, and 560 cm^–1^ may indeed be similarly weak, but they also fall outside the frequency range probed by Barlow et al.^25^ We are therefore able not only to assign peaks in our calculated spectra to all of the experimentally identified peaks (including revision of some previously tentative attributions) but also to explain why some simulated peaks are absent from the experimental data altogether. In higher-coverage experiments, further peaks arise at 1578 and 1630 cm^–1^, attributed by Barlow et al.^25^ to NH_2_ scissor and asymmetric CO_2_ stretch motions, respectively. These become active only because of significant changes to the binding geometry, where some molecules adopt a two-point attachment to the surface instead of the three-point attachment modeled here, but it is nevertheless noteworthy that our calculated peak at 1540 cm^–1^ contains precisely these components (alongside CO_2_ rock) and could readily be blue-shifted and split upon a change in conformation.

Overall, the correspondences listed in Table 2 reveal an rms discrepancy of 75 cm^–1^ between our AIMD results and the experimental peaks (falling to 26 cm^–1^ if we omit the poorly reproduced CH_2_ stretches). By way of comparison, we have conducted harmonic finite-displacement calculations of the vibrational spectrum (see the SI), obtaining an rms discrepancy over the same modes of 103 cm^–1^ (falling to 31 cm^–1^ omitting CH_2_ stretches). Viewed another way, our AIMD frequencies show an rms error of 3.2% relative to the corresponding experimental peaks (2.3% without CH_2_ modes), while our finite-displacement frequencies show an rms error of 4.2% (2.8% without CH_2_ modes). The anharmonic frequencies extracted from the AIMD simulation are thus in generally better agreement with the experiment than the harmonic frequencies derived from the finite-displacement method, both within and without the fingerprint region. We note, in passing, that it is common to apply variable scaling factors to harmonic vibrational frequencies in order to make closer contact with experimental results (see Sitathani et al.^41^ for a recent example in the context of surface species). We have not applied any such ad hoc correction to frequencies calculated within the current work; therefore, the improvement reported here genuinely reflects the inclusion of anharmonicity. Any remaining error is, therefore, most likely down either to the limited size of our simulation cell or to deficiencies inherent in our choice of exchange-correlation functional.

With the reliability of our results thus established for the 1/3 ML case, we may now confidently seek insight from comparison with the 1/6 ML results. For the most part, Table 1 and Figure 2 reveal that frequency shifts between the two different coverage regimes are generally fairly subtle within the fingerprint region. Among the high-frequency modes, in contrast, much more significant modification is evident. The two CH_2_ stretch modes found at 2950 and 3015 cm^–1^ in the low-coverage regime are blue-shifted by 30 and 55 cm^–1^, respectively, in the high-coverage case, while the low-coverage NH_2_ stretch modes found at 3440 and 3530 cm^–1^ are both red-shifted by 65 cm^–1^ at high coverage.

Most strikingly of all, however, we note that a small satellite peak of symmetric NH_2_ stretch character may be resolved at 3400 cm^–1^ in the low-coverage regime and that this is red-shifted by 120 cm^–1^ at high coverage. Alongside the modulation of the two main NH_2_ stretch peaks noted above, we believe this to be a clear signature of intermolecular hydrogen bonding. It is comparatively much harder to be certain about similar evidence from CO_2_ modes, of which only the wag varies by more than 20 cm^–1^ (from 700 cm^–1^ at 1/6 ML to 750 cm^–1^ at 1/3 ML) and even that is complicated by the involvement of NCC scissor motion. A peak featuring CO_2_ scissor and CC stretch occurs at 885 cm^–1^ in the low-coverage case and yet is not obviously present in the high-coverage case, but this is hardly convincing given the spectral complexity of this frequency region. Otherwise, the emergence of significant NH_2_ rocking and CN torsional motion at 1300 cm^–1^ in the high-coverage regime is perhaps attributable to hydrogen-bond formation, at least alongside the more diagnostic NH_2_ stretch evidence. Our calculated blue-shift in low-frequency CN and CC torsions (from 500 to 560 cm^–1^) might also be pertinent in this regard.

## Conclusions

In summary, we have shown that anharmonic vibrational frequencies derived from AIMD calculations are in slightly better agreement with experiment than are harmonic finite-displacement frequencies. Our spectra indicate that hydrogen bonds not only soften the NH_2_ stretch modes of adsorbed glycinate, as might have been expected, but also may stiffen the CN torsional mode and a portion of the NH_2_ rocking mode. Blue-shifts of the CH_2_ stretch modes provide an additional, albeit more indirect, indicator of the intermolecular interaction. Calculated thermal ellipsoids are significantly anisotropic and may be useful in benchmarking against future crystallographic analyses of overlayer structures. They are, moreover, dramatically altered upon incorporation of their molecule into a two-dimensional hydrogen-bonded network.

Finally, we affirm our belief that the methodologies employed in the present work ought to be more widely adopted in studies of surface systems likely to show strong anisotropy and/or anharmonicity in their vibrational motion. Much could be learnt, for instance, from a systematic survey of such effects in the many other amino acid overlayers that have been reported on various metal surfaces^32^ or in those aromatic adsorbates for which frustrated rotations are likely to be of more than passing interest.^42^

## Supporting Information Available

Notes on the analysis codes introduced in this work, additional technical details of density functional calculations and their analysis, commentary on the Wiener–Khintchine theorem, relationship between velocity power spectral density and the vibrational density of states, estimation of error in vibrational density of states, definition of the generalized local-mode coordinates adopted for glycinate in this work, details of calculated mean positions and numerical parameters defining calculated thermal ellipsoids, calculated harmonic normal modes (PDF)
