# Supplementary material for: First-Principles Simulation of Anharmonic and Anisotropic Vibrations of Glycinate on Copper
Source: ACS Omega. 2025 Feb 13;10(7):7422–7. doi: 10.1021/acsomega.5c00210 (PMC11866210; doi:10.1021/acsomega.5c00210)
Supplement: Supplementary file 1 — ao5c00210_si_001.pdf [file ao5c00210_si_001.pdf]

First-Principles Simulation of Anharmonic and Anisotropic Vibrations of Glycinate on Copper

Alexander D. Ievins
Yusuf Hamied Department of Chemistry, University of Cambridge, Lensfield Road, Cambridge, CB2 1EW, UK

Marco Sacchi
Department of Chemistry, University of Surrey, Guildford, GU2 7XH, UK

Stephen J. Jenkins
Yusuf HamiedDepartment of Chemistry, University of Cambridge, Lensfield Road, Cambridge, CB2 1EW, UK

A BRIEF NOTE ON NAMES

Trajectories obtained from the CASTEP computer code (as described below) were analysed using a suite of in-house computer codes named Calypso, Caliope and Capella. Respectively, these permit calculation of thermal ellipsoids, vibrational densities of states, and local vibrational modes. For the curious, the common first syllable of these names is intended to reflect not only their common origin in Cambridge, but also that all were written with analysis of CASTEP data in mind. Modification of these codes to work with output from other molecular dynamics codes ought to be essentially trivial. Beyond this, the names evoke a variety of mythological figures with a musical resonance, which we feel is apt for codes dealing with vibrations. The nymph Calypso enchanted Odysseus with her singing, keeping him prisoner for seven years, and it is this story, rather than the Carribean musical style, that we reference here; the name may be considered a near-homophone of the contraction "CA(STEP E)llipse" if one is willing to stretch a point. The nymph Caliope was famed in her own right as one of the muses, but also recognised as the mother of Orpheus, arguably the most well-known musical figure in all of mythology; a steam-driven musical instrument of the same name was once popular on riverboats and in circuses, where its unreliable tuning was matched only by the ambiguity of its pronunciation – we favour the four syllables of its original inspiration. Finally, the nymph Aega was nursemaid to the infant Zeus, at least in some tellings of the tale, and afterwards transformed into the star Capella; there is no etymological link whatsoever with the *a capella* style of music, but we could not resist the connotations of polyphony when naming a code that picks individual (local-mode) frequencies out of a crowd.

CALCULATIONAL DETAILS

First-principles density functional calculations were performed using the CASTEP computer code (Version 18.1) with a plane-wave kinetic energy cutoff at 340 eV [Clarke *et al*, *Z. Kristallogr.* **220**, 567-570 (2005)]. Electron-ion interactions were handled through the use of ultrasoft pseudopotentials [Vanderbilt, *Phys. Rev. B* **41**, 7892-7895 (1990)] and the exchange-correlation interactions through the Perdew-Wang form (PW91) of the generalised gradient approximation [Perdew *et al*, *Phys. Rev. B* **46**, 6671-6687 (1992)]. Electronic structure was converged with tolerances of 10^{-7} eV per atom in the energy and 10^{-3} eV.Å^{-1} in the forces. Sampling of the Brillouin zone was achieved with a 3×3×1 Monkhorst-Pack mesh [Monkhorst & Pack, *Phys. Rev. B* **13**, 5188-5192 (1976)] for a supercell whose cross-sectional dimensions were consistent with a (3×2) surface unit cell (at the theoretically obtained lattice constant of 3.6056 Å). The vertical dimension of the supercell was 20.40 Å, equivalent to 16 layers of the {110} surface. The surface itself was modelled by an eight-layer slab and all atoms were allowed to move in response to calculated forces.

Molecular dynamics calculations employed the NVT ensemble with target temperature 500 K, achieved via a five-link Nose-Hoover thermostat [Nosé, *J. Chem. Phys.* **81**, 511-519 (1984); Hoover, *Phys. Rev. A*, **31**, 1605-1697 (1985)] with characteristic ionic time set to 9.5 fs. The time-step for these calculations was 0.25 fs. Properties were calculated from the results of runs lasting 9 ps *after* discarding the first 1 ps to allow for equilibration.

In computing both total and local-mode vibrational densities of states (with the Caliope and Capella codes) power spectra were calculated (as defined in the following section) as the square modulus of a discrete Fourier transform of the system velocity. We down-sampled our data to a time-step of $\Delta t = 3.00$ fs to obtain a frequency resolution of $\Delta\omega = 3.49 \times 10^{-4}$ fs^{-1} ($\Delta f = 1.853$ cm^{-1}) over $N = 3000$ data-points. Gaussian broadening (with a standard deviation of 12 cm^{-1}) has been applied to the resulting spectra to suppress numerical noise while retaining meaningful details.

Thermal ellipsoids were calculated from our trajectories using the Calypso code, which provides output in the POV-Ray scene description language. The computed scene was then rendered using the open-source POV-Ray code (github.com/POV-Ray/povray) but this could equally have been done with any suitable ray-tracing package capable of reading the provided format (e.g. Blender). In line with the procedure defined by Kronenburg [*Acta. Cryst. A* **60**, 250-256 (2004)] ellipsoids are obtained by diagonalising the atomic displacement matrix for each atom in turn, scaling the resulting eigenvalues by a factor of 1.5958 so that the surface of each ellipsoid coincides with the expectation value of atomic displacement in every direction. Kronenburg [*ibid*] notes that a scaling factor of 1.5382 [B.T.M. Willis and A.W. Pryor, *Thermal Vibrations in Crystallography* (Cambridge University Press, 1975)] would result in ellipsoids that enclose precisely half the total probability of finding their corresponding atom at any given moment.

NOTES ON THE WIENER-KHINTCHINE THEOREM

The Wiener-Khintchine theorem [D.C. Champeney, *A Handbook of Fourier Theorems* (Cambridge University Press, 1987)] is a well-known result linking the power spectral density of a signal with the Fourier transform of its auto-correlation function. Since we rely upon it in calculating our vibrational density of states, we shall briefly recap its derivation in the context of the discrete time and frequency samplings employed in our work.

Let us begin by defining the discrete Fourier transform of a time-dependent complex variable $x[n\Delta t]$ as

$$x[k\Delta\omega] = \sum_{n=-N}^{N} x[n\Delta t]e^{-i(k\Delta\omega)(n\Delta t)} \tag{1}$$

where ω and t represent frequency and time respectively, with both k and n being integers. When dealing with a finite number of samples, $2N+1$, we shall consider that $x[n\Delta t]$ is periodic under the transformation $n \to n+2N+1$, without loss of generality. In practice, this is best achieved by sampling the time series over the range $n = [0, N]$ and then artificially imposing the mapping $x[-n\Delta t] = x[n\Delta t]$. This procedure neither adds nor subtracts any frequency components.

Note that the spectral resolution of this transform is determined by the duration of the time series, τ, such that $\Delta\omega = 2\pi/\tau$. From this, we deduce that $(2N+1)\Delta t\Delta\omega = 2\pi$, and hence that the exponential appearing in Eqn. 1 is also periodic under the transformation $n \to n+2N+1$ (and indeed under $k \to k+2N+1$ too).

Consider, then, the power spectral density of $x[n\Delta t]$, which we define as

$$\begin{aligned} S[k\Delta\omega] &= \lim_{N\to\infty} \frac{|x[k\Delta\omega]|^2}{2N+1} \\ &= \lim_{N\to\infty} \frac{1}{2N+1} \sum_{n'=-N}^{N} \sum_{n''=-N}^{N} x^*[n'\Delta t]x[n''\Delta t]e^{-i(k\Delta\omega)((n''-n')\Delta t)} \\ &= \lim_{N\to\infty} \frac{1}{2N+1} \sum_{n'=-N}^{N} \left[\sum_{n=-N-n'}^{N-n'} x^*[n'\Delta t]x[(n'+n)\Delta t]e^{-i(k\Delta\omega)(n\Delta t)} \right] \\ &= \lim_{N\to\infty} \frac{1}{2N+1} \sum_{n'=-N}^{N} \left[\sum_{n=-N-n'}^{N-n'} r_{n'}[n\Delta t]e^{-i(k\Delta\omega)(n\Delta t)} \right] \end{aligned} \tag{2}$$

where

$$r_{n'}[n\Delta t] = x^*[n'\Delta t]x[(n'+n)\Delta t] \tag{3}$$

and where the last two lines in Eqn. 2 have been written so as to emphasise the order in which the summations must be undertaken.

Now, given the periodicity of $x[n\Delta t]$, we can readily see that $r_{n'}[n\Delta t]$ is also periodic under the transformation $n \to n+2N+1$, and hence that

$$\sum_{n=-N-n'}^{N-n'} r_{n'}[n\Delta t]e^{-i(k\Delta\omega)(n\Delta t)} = \sum_{n=-N}^{N} r_{n'}[n\Delta t]e^{-i(k\Delta\omega)(n\Delta t)} \tag{4}$$

since the change in summation limits merely amounts to addressing the exact same summands in a different order.

In light of this observation, we are free to re-write our expression for the power spectral density as

$$\begin{aligned} S[k\Delta\omega] &= \lim_{N\to\infty} \frac{1}{2N+1} \sum_{n'=-N}^{N} \sum_{n=-N}^{N} r_{n'}[n\Delta t] e^{-i(k\Delta\omega)(n\Delta t)} \\ &= \lim_{N\to\infty} \sum_{n=-N}^{N} \left[\frac{1}{2N+1} \sum_{n'=-N}^{N} r_{n'}[n\Delta t] \right] e^{-i(k\Delta\omega)(n\Delta t)} \\ &= \lim_{N\to\infty} \sum_{n=-N}^{N} R[n\Delta t] e^{-i(k\Delta\omega)(n\Delta t)} \qquad (5) \end{aligned}$$

where we have made use of the independence of n and n' to reverse the order of summation, and where

$$\begin{aligned} R[n\Delta t] &= \frac{1}{2N+1} \sum_{n'=-N}^{N} r_{n'}[n\Delta t] \\ &= \frac{1}{2N+1} \sum_{n'=-N}^{N} x^*[n'\Delta t] x[(n'+n)\Delta t] \\ &= \langle x^*[0] x[n\Delta t] \rangle \qquad (6) \end{aligned}$$

is usually referred to as the autocorrelation function of the time series. The compact angle-bracket notation implies taking the average value of the enclosed expression for all $2N+1$ possible choices for "time zero" within the original periodic time series.

The Wiener-Khintchine theorem may thus simply be summarised by the statement that the power spectral density of a time series is equal to the Fourier transform of its autocorrelation function.

To this, we may add a useful corollary, relating to the summed power spectral density. Let us write this as

$$\begin{aligned} \lim_{N\to\infty} \sum_{k=0}^{2N} S[k\Delta\omega] &= \lim_{N\to\infty} \sum_{k=0}^{2N} \sum_{n=-N}^{N} R[n\Delta t] e^{-i(k\Delta\omega)(n\Delta t)} \\ &= \lim_{N\to\infty} \sum_{n=-N}^{N} R[n\Delta t] \left(\sum_{k=0}^{2N} e^{-i(k\Delta\omega)(n\Delta t)} \right) \\ &= R[0] \\ &= \langle |x[0]|^2 \rangle \qquad (7) \end{aligned}$$

in which we have made use of periodicity in the exponential. That is, the summed power spectral density is simply equivalent to the autocorrelation function at a delay time of zero.

VIBRATIONAL DENSITY OF STATES

It is commonly (and correctly) asserted that the vibrational density of states of an equilibrated system may be calculated by taking the Fourier transform of the velocity autocorrelation function (or equivalently, according to the Wiener-Khintchine theorem, the power spectral density of the velocity). Two potential sources of error or confusion exist, however, which are often skated over in the literature, and which would be worth clarifying. Firstly, whether the velocity (or its autocorrelation function) needs to be normalised in some fashion prior to taking its power spectrum (or Fourier transform) and secondly, whether the velocity should be mass-weighted or not. In this work, we apply *no* pre-normalisation but *do* use mass-weighted velocities. Here we confirm that this leads to a correct result, drawing heavily upon the argument set out by M.T. Dove in his *Introduction to Lattice Dynamics* (Cambridge University Press, 1993).

Let us start by denoting as $Q^{\nu}_{j\alpha}$ the mass-weighted displacement amplitude of the atom labelled j, in the Cartesian direction labelled α, when oscillating as part of the harmonic normal mode labelled ν. Such a quantity is simply equivalent to the corresponding non-weighted displacement amplitude multiplied by $\sqrt{m_j}$ (i.e. the square root of the atom's mass). Since we are treating our system as harmonic, the instantaneous mass-weighted displacements in this mode take the form

$$q^{\nu}_{j\alpha} = Q^{\nu}_{j\alpha} \sin\left(\omega_\nu t + \phi_\nu\right) \tag{8}$$

where ω_ν is the mode's angular frequency, ϕ_ν is a phase offset, and t represents time. The mass-weighted velocities for this mode are then given by

$$v^{\nu}_{j\alpha} = \omega_\nu Q^{\nu}_{j\alpha} \cos\left(\omega_\nu t + \phi_\nu\right) \tag{9}$$

upon differentiating with respect to the time variable. Note that we are implicitly limiting ourselves to zone-centre oscillations, for which the amplitudes will necessarily be real. Generalisation is essentially straightforward, but superfluous to our present purpose. We shall furthermore insist that the mass-weighted normal-mode amplitudes are orthonormal, so that

$$\sum_j \sum_\alpha Q^{\nu}_{j\alpha} Q^{\nu'}_{j\alpha} = \delta_{\nu\nu'} \sum_j \sum_\alpha (Q^{\nu}_{j\alpha})^2 \tag{10}$$

where $\delta_{\nu\nu'}$ is the Kronecker delta.

With these definitions, we may write the instantaneous kinetic energy of mode ν as

$$\frac{1}{2} \sum_j \sum_\alpha (v^{\nu}_{j\alpha})^2 = \frac{1}{2} \sum_j \sum_\alpha (\omega_\nu Q^{\nu}_{j\alpha})^2 \cos^2\left(\omega_\nu t + \phi_\nu\right) \tag{11}$$

and hence its time-averaged kinetic energy as

$$\frac{1}{4} \sum_j \sum_\alpha (\omega_\nu Q^{\nu}_{j\alpha})^2 \tag{12}$$

since the mean of a squared cosine function is always one half. At equilibrium, equipartition implies that this last quantity ought simply to equal $k_B T/2$, yielding

$$\sum_j \sum_\alpha (\omega_\nu Q^{\nu}_{j\alpha})^2 = 2 k_B T \tag{13}$$

as a convenient result to carry forward into our analysis of multi-modal oscillation.

When the system oscillates in a combination of its normal modes, the instantaneous mass-weighted displacements of its atoms are simply additive, allowing us to write

$$\begin{aligned} q_{j\alpha} &= \sum_{\nu} q^{\nu}_{j\alpha} \\ &= \sum_{\nu} Q^{\nu}_{j\alpha} \sin\left(\omega_{\nu} t + \phi_{\nu}\right) \end{aligned} \tag{14}$$

and hence to obtain

$$v_{j\alpha} = \sum_{\nu} \omega_{\nu} Q^{\nu}_{j\alpha} \cos\left(\omega_{\nu} t + \phi_{\nu}\right) \tag{15}$$

as the mass-weighted velocity for atom j in direction α.

With this, we now construct the velocity autocorrelation function

$$\begin{aligned} \sum_{j}\sum_{\alpha}\langle v^{*}_{j\alpha}(0) v_{j\alpha}(t)\rangle &= \sum_{j}\sum_{\alpha}\langle v_{j\alpha}(0) v_{j\alpha}(t)\rangle \\ &= \sum_{j}\sum_{\alpha}\langle \sum_{\nu}\sum_{\nu'} \omega_{\nu}\omega_{\nu'} Q^{\nu}_{j\alpha} Q^{\nu'}_{j\alpha} \cos\phi_{\nu}\cos\left(\omega_{\nu'} t + \phi_{\nu'}\right)\rangle \\ &= \sum_{\nu}\sum_{\nu'} \omega_{\nu}\omega_{\nu'} \langle \cos\phi_{\nu}\cos\left(\omega_{\nu'} t + \phi_{\nu'}\right)\rangle \sum_{j}\sum_{\alpha} Q^{\nu}_{j\alpha} Q^{\nu'}_{j\alpha} \\ &= \sum_{\nu}\sum_{\nu'} \omega_{\nu}\omega_{\nu'} \langle \cos\phi_{\nu}\cos\left(\omega_{\nu'} t + \phi_{\nu'}\right)\rangle \delta_{\nu\nu'} \sum_{j}\sum_{\alpha} (Q^{\nu}_{j\alpha})^2 \\ &= \sum_{\nu} \langle \cos\phi_{\nu}\cos\left(\omega_{\nu} t + \phi_{\nu}\right)\rangle \sum_{j}\sum_{\alpha} (\omega^2_{\nu} Q^{\nu}_{j\alpha})^2 \\ &= 2k_B T \sum_{\nu} \langle \cos\phi_{\nu}\cos\left(\omega_{\nu} t + \phi_{\nu}\right)\rangle \end{aligned} \tag{16}$$

where we have invoked the orthogonality of the normal-mode coordinates, and made use of the expression for the squared normal-mode coordinates derived above from the equipartition theorem (Eqn. 13).

Now, the term within the angle brackets may be expanded through standard trigonometry, allowing us to write

$$\begin{aligned} \langle \cos\phi_{\nu}\cos\left(\omega_{\nu} t + \phi_{\nu}\right)\rangle &= \langle \cos\phi_{\nu}(\cos\omega_{\nu} t\cos\phi_{\nu} - \sin\omega_{\nu} t \sin\phi_{\nu})\rangle \\ &= \langle \cos\omega_{\nu} t \cos^2\phi_{\nu} - \sin\omega_{\nu} t \sin\phi_{\nu}\cos\phi_{\nu}\rangle \\ &= \cos\omega_{\nu} t\langle \cos^2\phi_{\nu}\rangle - \sin\omega_{\nu} t\langle \sin\phi_{\nu}\cos\phi_{\nu}\rangle \end{aligned} \tag{17}$$

in which the angle brackets may now be interpreted as averaging over all possible values of ϕ_{ν}. In this manner, we readily obtain $\langle \cos^2\phi_{\nu}\rangle = 1/2$ and $\langle \sin\phi_{\nu}\cos\phi_{\nu}\rangle = 0$, giving us

$$\langle \cos\phi_{\nu}\cos\left(\omega_{\nu} t + \phi_{\nu}\right)\rangle = \frac{1}{2}\cos\omega_{\nu} t \tag{18}$$

and finally allowing us to write

$$\sum_{j}\sum_{\alpha}\langle v^{*}_{j\alpha}(0) v_{j\alpha}(t)\rangle = k_B T \sum_{\nu} \cos\omega_{\nu} t \tag{19}$$

for the autocorrelation function. It therefore follows that the Fourier transform of the autocorrelation function will comprise a single delta-function peak at each normal-mode frequency, each integrating to the same intensity.

In fact, this justifies us in identifying the vibrational density of states (normalised to correspond to a total of N modes) as

$$g(\omega) = \frac{1}{k_B T} \mathcal{F}\left(\sum_j \sum_\alpha \langle v^*_{j\alpha}(0) v_{j\alpha}(t) \rangle \right) = \frac{1}{k_B T} \sum_j \sum_\alpha \mathcal{F}\left(\langle v^*_{j\alpha}(0) v_{j\alpha}(t) \rangle \right) \tag{20}$$

and by dint of the Wiener-Khintchine theorem this may also be expressed in terms of the power spectral density of the individual mass-weighted velocity components

$$g(\omega) = \frac{1}{k_B T} \sum_j \sum_\alpha \mathcal{P}\left(v_{j\alpha}(t) \right) \tag{21}$$

where $\mathcal{F}$ implies taking the Fourier transform, and $\mathcal{P}$ the power spectrum. Note that removal of mass weighting from the velocity components prior to construction of the autocorrelation function or power spectral density would invalidate the above analysis. It is therefore essential that mass-weighted velocities be used, but unnecessary to apply any other normalisation to these velocities (or their autocorrelation function) prior to taking their power spectrum (or Fourier transform).

ESTIMATION OF ERROR IN THE VIBRATIONAL DENSITY OF STATES

Vibrational density of states plots presented in the paper were derived from 9 ps simulations, which should be long enough for even frustrated translational and rotational modes to execute in the order of ten complete cycles. Modes within the fingerprint region will execute hundreds of cycles, and the very highest-frequency modes will approach one thousand cycles. We believe, therefore, that there is good cause to anticipate well-equilibrated results, but it will nevertheless be wise to test this expectation. To that end, we have divided the full simulation time (for each coverage) into three consecutive subsets of 3 ps duration. For each of these, we compute the power spectral density and at each separate frequency we calculate both the mean and the standard deviation. We then plot the mean for each system as the red curves in Fig. S1, noting that these are almost indistinguishable from the 9 ps results plotted in the paper. The surrounding grey region in each case represents values lying (for each frequency) within the standard error in the mean, which is given by the standard deviation divided by the square root of the number of samples (three samples in this case). This uncertainty envelope is rather smaller for the 1/3 ML simulation compared with the 1/6 ML simulation, owing to the more tightly constrained molecular motion in the higher-coverage overlayer, but in either case it is sufficiently small that the features we discuss are not in doubt.

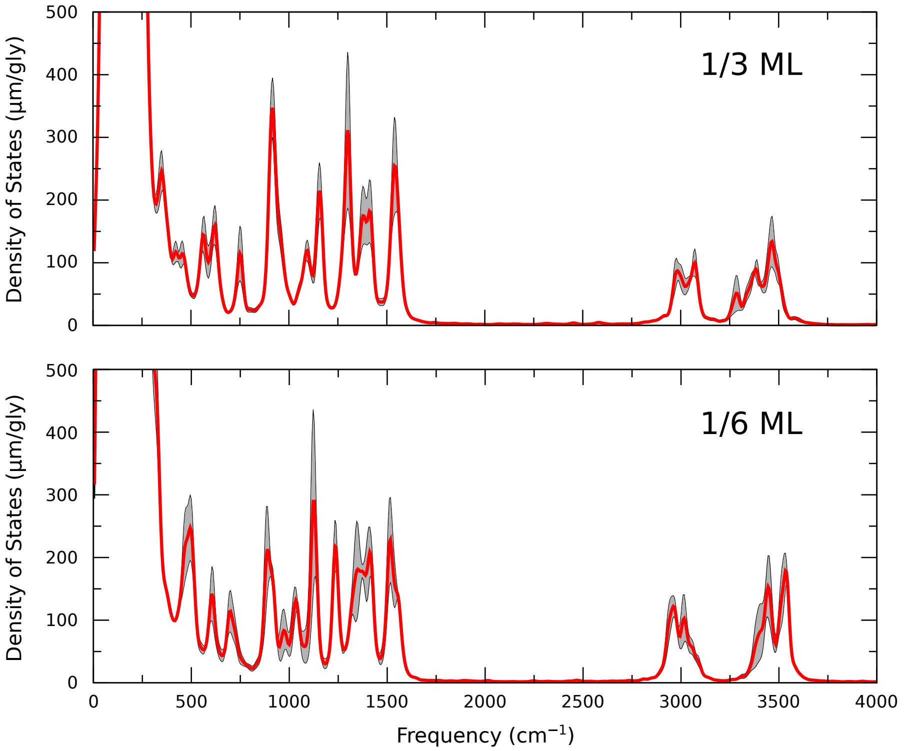

FIG. S1. Calculated vibrational density of states for 1/3 ML and 1/6 ML glycinate on Cu{110}. In each case, the red curve shows the mean obtained from three consecutive 3 ps segments of the full 9 ps simulation, while the range of the surrounding grey region indicates the standard error in the mean.

Further insight may be gleaned from Fig. S2, where a comparison is made between the density of states curves presented in Fig. 2 of the paper and the mean density of states obtained from nine independent 9 ps simulations of the gas-phase glycine anion (calculated on essentially identical terms to the surface calculations, albeit using only a single k-point sampling of the Brillouin zone). That is to say, the gas-phase curves represent almost an order of magnitude greater integration time than the surface curves. Nevertheless, it is clear that the surface curves show very little evidence of any systematic lack of weight in any region of the spectrum, which might have been indicative of poor equilibration. The point is made yet more forcefully in Fig. S3, where cumulative plots representing the total number of states up to a particular frequency show the same broad shape between adsorbed and free glycinate. Differences of detail are, of course, to be expected, otherwise there would be no need for the surface calculations.

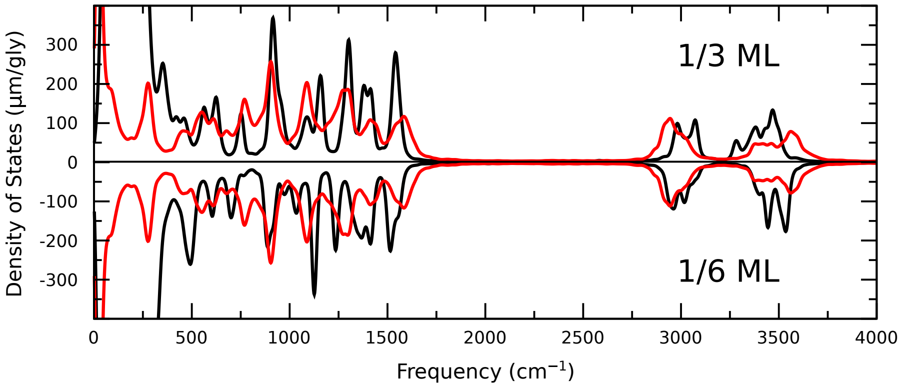

FIG. S2. Calculated vibrational density of states for 1/3 ML and 1/6 ML glycinate on Cu{110}. In each case, the black curve shows the density of states obtained from the full 9 ps simulation of the relevant surface system, while the red curve shows the mean density of states from nine independent 9 ps simulations of the gas-phase glycinate anion.

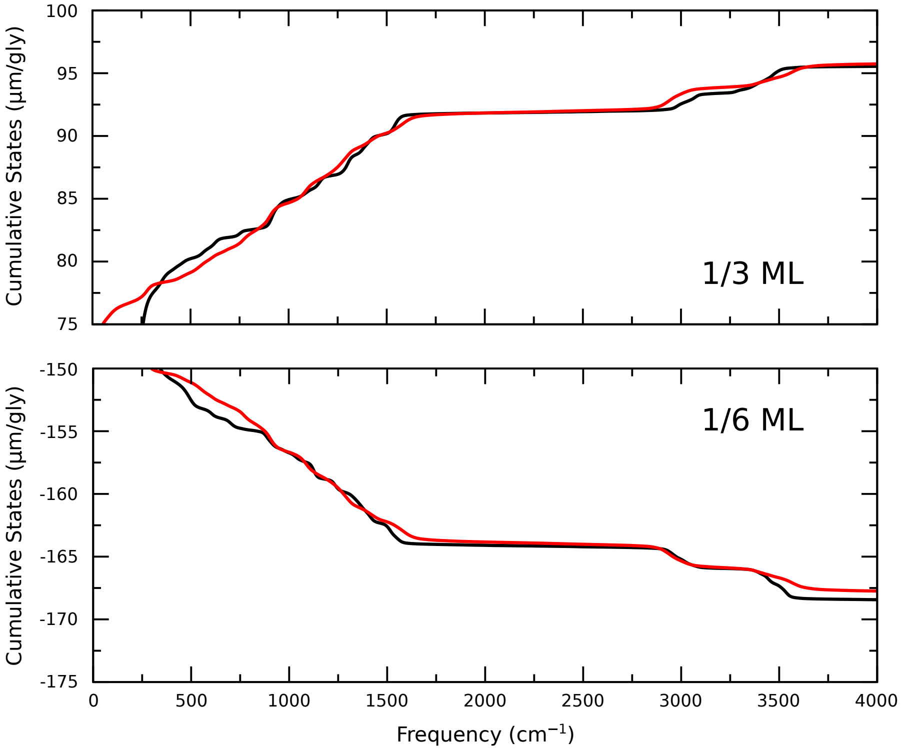

FIG. S3. Cumulative plots of the density of states data from Fig. S2, which is to say running integrals of the area under each curve. The traces for the gas-phase anion have been shifted by 72 states in the upper plot, and by 144 states in the lower plot, to reflect the additional states (per glycinate moiety) due to the substrate.

DEFINITION OF GENERALISED LOCAL-MODE COORDINATES

For the analysis of local-mode contributions to the overall vibrational density of states, we adopt a system of generalised coordinates adapted from those proposed by Shimanouchi (in the general case) [Tables of Molecular Vibrational Frequencies Consolidated, Vol I. National Bureau of Standards, 1972] and by Vijay and Sathyanarayana (specifically for glycine) [*J. Phys. Chem.* **96**, 10735-10739 (1992)]. For clarity, we first establish a numbering convention for atoms within the adsorbed molecule (Fig. S4) and then define a set of vectors $\mathbf{R}_{ab}$ linking atom a to atom b for chemically bonded pairs, with $\hat{\mathbf{R}}_{ab}$ denoting the corresponding unit vector in each case. Bond angles may then be defined as $\theta_{abc} = \cos^{-1}[\hat{\mathbf{R}}_{ba}.\hat{\mathbf{R}}_{bc}]$. That is, for example, the symbol θ_{172} represents the angle formed between the two N–H bonds meeting at the nitrogen atom, while θ_{657} represents that between the C–C and C–N bonds meeting at the central carbon atom.

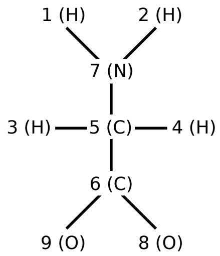

FIG. S4. Numbering convention for atoms within a glycinate moiety.

With these in place, it is possible to define fifteen of the twenty-one generalised coordinates tabulated on the following page. For the remainder, however, it will prove convenient to establish two systems of Cartesian axes based upon the planes created on the one hand by the NH_2 group and on the other by the CO_2 group. We start by defining

$$\hat{\mathbf{Z}}_{1725} = (\mathbf{R}_{71} \times \mathbf{R}_{72})/|\mathbf{R}_{71} \times \mathbf{R}_{72}| \tag{22}$$
$$\hat{\mathbf{Z}}_{6895} = (\mathbf{R}_{68} \times \mathbf{R}_{69})/|\mathbf{R}_{68} \times \mathbf{R}_{69}| \tag{23}$$

to be unit vectors normal to the NH_2 and CO_2 planes, respectively, and then define

$$\hat{\mathbf{Y}}_{1725} = (\mathbf{R}_{75} \times \hat{\mathbf{Z}}_{172})/|\mathbf{R}_{75} \times \hat{\mathbf{Z}}_{172}| \tag{24}$$
$$\hat{\mathbf{Y}}_{6895} = (\mathbf{R}_{65} \times \hat{\mathbf{Z}}_{869})/|\mathbf{R}_{65} \times \hat{\mathbf{Z}}_{869}| \tag{25}$$

to be unit vectors lying within the plane of the relevant group but perpendicular to the bond linking that group to the rest of the molecule. Finally, we complete the set with

$$\hat{\mathbf{X}}_{1725} = (\hat{\mathbf{Y}}_{1725} \times \hat{\mathbf{Z}}_{1725}) \tag{26}$$
$$\hat{\mathbf{X}}_{6895} = (\hat{\mathbf{Y}}_{8695} \times \hat{\mathbf{Z}}_{8695}) \tag{27}$$

and note that the following vectors

$$\hat{\mathbf{V}}_{1725} = (\hat{\mathbf{Y}}_{1725} \times \hat{\mathbf{R}}_{75}) \tag{28}$$
$$\hat{\mathbf{V}}_{6895} = (\hat{\mathbf{Y}}_{8695} \times \hat{\mathbf{R}}_{65}) \tag{29}$$
$$\hat{\mathbf{W}}_{657} = (\mathbf{R}_{56} \times \mathbf{R}_{57})/|\mathbf{R}_{56} \times \mathbf{R}_{57}| \tag{30}$$
$$\hat{\mathbf{W}}_{756} = (\mathbf{R}_{57} \times \mathbf{R}_{56})/|\mathbf{R}_{57} \times \mathbf{R}_{56}| \tag{31}$$

will turn out to be useful in calculating torsional angles.

In terms of these vectors and angles, the generalised coordinates may be summarised as follows...

S_1	$= \lvert\mathbf{R}_{71}\rvert + \lvert\mathbf{R}_{72}\rvert$	$\nu_s(NH_2)$	Symmetric Stretch
S_2	$= \lvert\mathbf{R}_{57}\rvert$	$\nu(CN)$	Stretch
S_3	$= \lvert\mathbf{R}_{56}\rvert$	$\nu(CC)$	Stretch
S_4	$= \lvert\mathbf{R}_{53}\rvert + \lvert\mathbf{R}_{54}\rvert$	$\nu_s(CH_2)$	Symmetric Stretch
S_5	$= \lvert\mathbf{R}_{68}\rvert + \lvert\mathbf{R}_{69}\rvert$	$\nu_s(CO_2)$	Symmetric Stretch
S_6	$= \theta_{172}$	$\delta(NH_2)$	Scissor
S_7	$= \mathrm{sgn}[\hat{\mathbf{Z}}_{1725}.\hat{\mathbf{R}}_{57}] \cos^{-1}[\hat{\mathbf{X}}_{1725}.\hat{\mathbf{R}}_{57}]$	$\omega(NH_2)$	Wag
S_8	$= 4\theta_{354} - \theta_{356} - \theta_{456} - \theta_{357} - \theta_{457}$	$\delta(CH_2)$	Scissor
S_9	$= \theta_{356} + \theta_{456} - \theta_{357} - \theta_{457}$	$\omega(CH_2)$	Wag
S_{10}	$= \theta_{869}$	$\delta(CO_2)$	Scissor
S_{11}	$= \mathrm{sgn}[\hat{\mathbf{Z}}_{6895}.\hat{\mathbf{R}}_{56}] \cos^{-1}[\hat{\mathbf{X}}_{6895}.\hat{\mathbf{R}}_{56}]$	$\omega(CO_2)$	Wag
S_{12}	$= 5\theta_{756} - \theta_{356} - \theta_{456} - \theta_{357} - \theta_{457} - \theta_{354}$	$\delta(NCC)$	Scissor
S_{13}	$= \lvert\mathbf{R}_{71}\rvert - \lvert\mathbf{R}_{72}\rvert$	$\nu_a(NH_2)$	Antisymmetric Stretch
S_{14}	$= \lvert\mathbf{R}_{53}\rvert - \lvert\mathbf{R}_{54}\rvert$	$\nu_a(CH_2)$	Antisymmetric Stretch
S_{15}	$= \lvert\mathbf{R}_{68}\rvert - \lvert\mathbf{R}_{69}\rvert$	$\nu_a(CO_2)$	Antisymmetric Stretch
S_{16}	$= \mathrm{sgn}[\hat{\mathbf{Y}}_{1725}.\hat{\mathbf{R}}_{71}] \cos^{-1}[\hat{\mathbf{X}}_{1725}.\hat{\mathbf{R}}_{71}] + \mathrm{sgn}[\hat{\mathbf{Y}}_{1725}.\hat{\mathbf{R}}_{72}] \cos^{-1}[\hat{\mathbf{X}}_{1725}.\hat{\mathbf{R}}_{72}]$	$\rho(NH_2)$	Rock
S_{17}	$= \theta_{356} - \theta_{456} + \theta_{357} - \theta_{457}$	$\rho(CH_2)$	Rock
S_{18}	$= \mathrm{sgn}[\hat{\mathbf{Y}}_{8695}.\hat{\mathbf{R}}_{68}] \cos^{-1}[\hat{\mathbf{X}}_{8695}.\hat{\mathbf{R}}_{68}] + \mathrm{sgn}[\hat{\mathbf{Y}}_{8695}.\hat{\mathbf{R}}_{69}] \cos^{-1}[\hat{\mathbf{X}}_{8695}.\hat{\mathbf{R}}_{69}]$	$\rho(CO_2)$	Rock
S_{19}	$= \theta_{356} - \theta_{456} - \theta_{357} + \theta_{457}$	$t(CH_2)$	Twist
S_{20}	$= \mathrm{sgn}[\hat{\mathbf{W}}_{657}.\hat{\mathbf{V}}_{1725}] \cos^{-1}[\hat{\mathbf{W}}_{657}.\hat{\mathbf{Y}}_{1725}]$	$\tau(CN)$	Torsion
S_{21}	$= \mathrm{sgn}[\hat{\mathbf{W}}_{756}.\hat{\mathbf{V}}_{8695}] \cos^{-1}[\hat{\mathbf{W}}_{756}.\hat{\mathbf{Y}}_{8695}]$	$\tau(CC)$	Torsion

Amongst these, six relate purely to motion of the CH_2 group (two stretches, scissor, wag, rock and twist) and are defined exactly as in the work of Vijay and Sathyanarayana [*ibid.*] in their study of glycine. The CN and CC stretches are also defined in line with that work, as is the NCC scissor coordinate.

For the NH_2 group, three generalised coordinates (two stretches and scissor) involve motion purely within the plane of the three constituent atoms, and these we define exactly as per the previous glycine study [*ibid.*]. In addition, however, we also define a fourth generalised coordinate (rock) to be confined to this plane, and in this we differ from the prior work. Specifically, we take $\cos^{-1}[\hat{\mathbf{X}}_{1725}.\hat{\mathbf{R}}_{71}]$ to measure the angle between the $\mathbf{R}_{71}$ bond and one of the in-plane reference axes defined above. Multiplication by $\mathrm{sgn}[\hat{\mathbf{Y}}_{1725}.\hat{\mathbf{R}}_{71}]$ then ensures the correct sign convention for anticlockwise (positive) and clockwise (negative) rotation when viewed from the $\hat{\mathbf{Z}}_{1725}$ direction. A similar expression yields the equivalent information for the $\mathbf{R}_{72}$ bond, and the sum of both angles then measures the overall angular deflection of NH_2 within its plane, independent of any stretch or scissor motion that may also occur.

As for the NH_2 wag coordinate, we again differ from previous work [*ibid.*] by defining this explicitly in terms of the plane defined by the group's three constituent atoms, in which we are inspired by the approach taken by Shimanouchi [*op. cit.*] when dealing with a three-fold coordinated carbon atom. In this instance, we take $\cos^{-1}[\hat{\mathbf{X}}_{1725}.\hat{\mathbf{R}}_{57}]$ to measure the angular deflection of this plane relative to the direction of the back-bond linking the group to the rest of the molecule. Multiplication by $\mathrm{sgn}[\hat{\mathbf{Z}}_{1725}.\hat{\mathbf{R}}_{57}]$ then ensures that the generalised coordinate changes sign either side of its zero point, which coincides with the geometry where the back-bond lies within the plane. In similar fashion, we take $\cos^{-1}[\hat{\mathbf{W}}_{657}.\hat{\mathbf{Y}}_{1725}]$ to measure torsion around the CN bond, multiplied by $\mathrm{sgn}[\hat{\mathbf{W}}_{657}.\hat{\mathbf{V}}_{1725}]$ to ensure an appropriate change of sign when passing through the zero point.

Finally, for the CO_2 group, we necessarily diverge again from Vijay and Sathyanarayana [*ibid.*] because our molecule lacks one hydrogen atom compared with theirs. Not only does this imply three fewer generalised coordinates in total, but also that the carboxylate group can (and should) be treated on the same basis as the NH_2 group. Our expressions for CO_2 are thus exactly analogous to those defined above, replacing $1 \rightarrow 8$, $2 \rightarrow 9$ and $7 \rightarrow 6$ to achieve the correct structural equivalence.

MEAN POSITIONS AND THERMAL ELLIPSOIDS

In the paper, thermal ellipsoids for the 1/3 ML coverage were presented after the imposition of an explicit glide symmetry, while those for 1/6 ML coverage could only be used in their raw state. For completeness, we here show how little the symmetrisation procedure actually affects the 1/3 ML results (Fig. S5). We also tabulate non-symmetrised and symmetrised data for the 1/3 ML calculations (Tables S1 and S2) as well as for the 1/6 ML calculations (Table S3). Positions are given in Cartesian coordinates and the surface model was periodic over 7.649 Å in the $\hat{\mathbf{x}}$ direction and 7.211 Å in the $\hat{\mathbf{y}}$ direction. Eigenvalues of the atomic displacement matrix defined in the main text are presented here as λ_n, and the corresponding normalised eigenvectors, w_n, are given in row format.

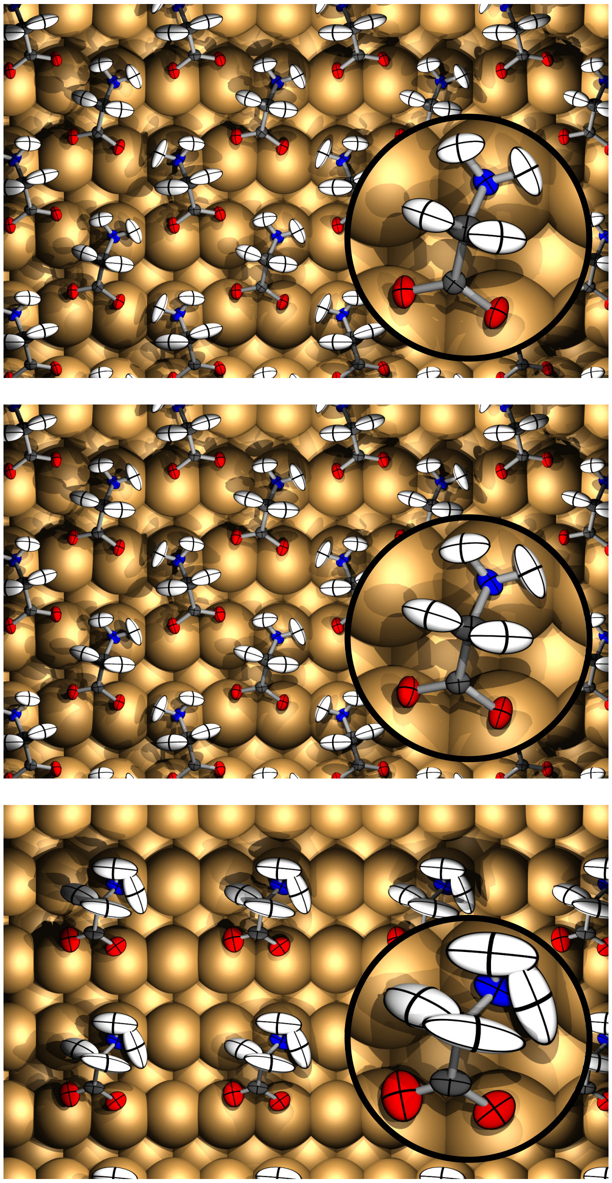

FIG. S5. Thermal ellipsoids calculated at 500 K for glycinate on Cu{110}. The upper panel was created using unsymmetrised data direct from the 1/3 ML simulation, while the middle panel was created from the same data but imposing glide symmetry upon the displacement matrices prior to diagonalisation. Differences are barely perceptible to the eye, but mainly affect the NH_2 groups. The lower panel shows results from the 1/6 ML simulation for comparison. White, grey, blue and red ellipsoids indicate H, C, N and O atoms, while Cu atoms are represented as just-larger-than-touching spheres (not thermal ellipsoids).

TABLE S1. Non-symmetrised mean positions and non-symmetrised thermal ellipsoid parameters calculated for glycinate at 1/3 ML coverage (used in creating the upper panel in Fig. S5).

Atom	Mean Position (Å)	$1.5958\sqrt{\lambda}$ (Å)	$\mathbf{w}_n$ (dimensionless)
H	(0.798, 1.735, 11.806)	0.722	(0.407, 0.910, -0.080)
		0.345	(0.508, -0.153, 0.848)
		0.245	(0.759, -0.386, -0.524)
H	(2.310, 2.212, 11.730)	0.652	(0.918, -0.057, -0.393)
		0.358	(-0.090, 0.935, -0.344)
		0.259	(0.387, 0.351, 0.853)
H	(1.391, 0.166, 13.120)	0.954	(0.815, -0.005, 0.580)
		0.373	(0.009, 0.999, -0.021)
		0.271	(-0.580, 0.022, 0.815)
H	(2.973, 0.671, 12.960)	0.844	(0.889, 0.229, -0.397)
		0.337	(-0.337, 0.913, -0.228)
		0.321	(0.310, 0.336, 0.889)
H	(5.729, 5.872, 11.712)	0.578	(-0.939, -0.209, -0.273)
		0.362	(-0.053, 0.873, -0.484)
		0.245	(-0.339, 0.440, 0.831)
H	(7.258, 5.335, 11.793)	0.604	(-0.452, 0.892, 0.014)
		0.329	(-0.736, -0.382, 0.560)
		0.252	(-0.505, -0.242, -0.829)
H	(4.973, 4.309, 12.908)	0.730	(-0.789, 0.268, -0.554)
		0.354	(0.345, 0.938, -0.038)
		0.261	(-0.509, 0.221, 0.832)
H	(6.577, 3.802, 13.158)	0.755	(-0.883, 0.093, 0.459)
		0.380	(0.055, 0.994, -0.095)
		0.246	(0.466, 0.058, 0.883)
C	(2.136, 0.373, 12.444)	0.504	(0.968, 0.091, 0.236)
		0.291	(-0.044, 0.978, -0.197)
		0.205	(-0.249, 0.180, 0.952)
C	(2.445, 6.340, 11.666)	0.295	(0.979, 0.055, 0.196)
		0.262	(-0.057, 0.998, 0.003)
		0.185	(-0.195, -0.014, 0.981)
C	(5.851, 4.003, 12.432)	0.413	(-0.981, 0.190, -0.030)
		0.303	(0.192, 0.971, -0.144)
		0.168	(-0.002, 0.147, 0.989)
C	(5.578, 2.750, 11.651)	0.280	(0.611, 0.792, 0.001)
		0.271	(-0.788, 0.608, -0.102)
		0.153	(-0.081, 0.062, 0.995)
N	(1.713, 1.456, 11.596)	0.383	(-0.577, -0.739, 0.347)
		0.264	(0.787, -0.390, 0.478)
		0.194	(-0.218, 0.549, 0.807)
N	(6.316, 5.085, 11.576)	0.332	(0.573, -0.779, 0.254)
		0.271	(-0.819, -0.551, 0.157)
		0.192	(-0.017, 0.298, 0.954)
O	(3.615, 6.143, 11.291)	0.382	(-0.335, 0.781, -0.527)
		0.303	(-0.065, 0.539, 0.840)
		0.264	(0.940, 0.315, -0.130)
O	(1.476, 5.608, 11.355)	0.385	(-0.199, 0.958, -0.208)
		0.259	(0.967, 0.226, 0.115)
		0.196	(-0.157, 0.178, 0.971)
O	(6.565, 2.039, 11.352)	0.400	(0.363, 0.874, -0.322)
		0.261	(-0.904, 0.247, -0.350)
		0.236	(-0.227, 0.418, 0.880)
O	(4.413, 2.523, 11.266)	0.387	(-0.016, 0.953, -0.302)
		0.277	(-0.956, -0.103, -0.275)
		0.239	(-0.293, 0.284, 0.913)

TABLE S2. Non-symmetrised mean positions and symmetrised thermal ellipsoid parameters calculated for glycinate at 1/3 ML coverage (used in creating the middle panel in Fig. S5).

Atom	Mean Position (Å)	$1.5958\sqrt{\lambda}$ (Å)	$\mathbf{w}_n$ (dimensionless)
H	(0.798, 1.735, 11.806)	0.665	(0.425, 0.904, -0.042)
		0.335	(0.623, -0.258, 0.739)
		0.257	(-0.657, 0.340, 0.673)
H	(2.310, 2.212, 11.730)	0.614	(0.931, -0.118, -0.346)
		0.363	(-0.041, 0.906, -0.422)
		0.253	(0.363, 0.407, 0.838)
H	(1.391, 0.166, 13.120)	0.858	(0.843, 0.036, 0.536)
		0.378	(0.005, 0.997, -0.074)
		0.264	(-0.537 0.065, 0.841)
H	(2.973, 0.671, 12.960)	0.786	(0.850, 0.245, -0.467)
		0.347	(-0.342, 0.930, -0.135)
		0.299	(0.401, 0.275, 0.874)
H	(5.729, 5.872, 11.712)	0.614	(-0.931, -0.118, -0.346)
		0.363	(0.041, 0.906, -0.422)
		0.253	(-0.363, 0.407, 0.838)
H	(7.258, 5.335, 11.793)	0.665	(-0.425, 0.904, -0.042)
		0.335	(-0.623, -0.258, 0.739)
		0.257	(0.657, 0.340, 0.673)
H	(4.973, 4.309, 12.908)	0.786	(-0.850, 0.245, -0.467)
		0.347	(0.342, 0.930, -0.135)
		0.299	(-0.401, 0.275, 0.874)
H	(6.577, 3.802, 13.158)	0.858	(-0.843, 0.036, 0.536)
		0.378	(-0.005, 0.997, -0.074)
		0.264	(0.537, 0.065, 0.841)
C	(2.136, 0.373, 12.444)	0.457	(0.984, 0.122, 0.132)
		0.298	(-0.095, 0.977, -0.189)
		0.195	(-0.152, 0.174, 0.973)
C	(2.445, 6.340, 11.666)	0.283	(0.991, -0.115, 0.064)
		0.269	(0.117, 0.993, -0.019)
		0.173	(-0.061, 0.027, 0.998)
C	(5.851, 4.003, 12.432)	0.457	(-0.984, 0.122, 0.132)
		0.298	(0.095, 0.977, -0.189)
		0.195	(0.152, 0.174, 0.973)
C	(5.578, 2.750, 11.651)	0.283	(-0.991, -0.115, 0.064)
		0.269	(-0.117, 0.993, -0.019)
		0.173	(0.061, 0.027, 0.998)
N	(1.713, 1.456, 11.596)	0.358	(0.580, 0.754, -0.309)
		0.265	(0.810, -0.494, 0.316)
		0.196	(-0.086, 0.433, 0.897)
N	(6.316, 5.085, 11.576)	0.358	(-0.580, 0.754, -0.309)
		0.265	(-0.810, -0.494, 0.316)
		0.196	(0.086, 0.433, 0.897)
O	(3.615, 6.143, 11.291)	0.380	(-0.161, 0.908, -0.385)
		0.278	(-0.466, 0.274, 0.841)
		0.273	(0.870, 0.315, 0.379)
O	(1.476, 5.608, 11.355)	0.391	(-0.291, 0.918, -0.267)
		0.260	(0.954, 0.300, -0.009)
		0.220	(-0.072, 0.258, 0.964)
O	(6.565, 2.039, 11.352)	0.391	(0.291, 0.918, -0.267)
		0.260	(-0.954, 0.300, -0.009)
		0.220	(0.072, 0.258, 0.964)
O	(4.413, 2.523, 11.266)	0.380	(0.161, 0.908, -0.385)
		0.278	(0.466, 0.274, 0.841)
		0.273	(-0.870, 0.315, 0.379)

TABLE S3. Mean positions and thermal ellipsoid parameters calculated for glycinate at 1/6 ML coverage (used in creating the lower panel in Fig. S5).

Atom	Mean Position (Å)	$1.5958\sqrt{\lambda}$ (Å)	$\mathbf{w}_n$ (dimensionless)
H	(6.374, 5.746, 12.147)	1.059	(0.978, -0.086, 0.192)
		0.498	(0.102, 0.991, -0.080)
		0.353	(-0.183, 0.098, 0.978)
H	(7.309, 4.657, 11.976)	1.068	(-0.347, 0.902, 0.257)
		0.490	(0.868, 0.412, -0.278)
		0.323	(0.357, -0.127, 0.926)
H	(4.750, 4.515, 12.566)	1.172	(0.682, -0.281, 0.676)
		0.551	(0.651, 0.655, -0.384)
		0.385	(-0.335, 0.701, 0.630)
H	(5.948, 3.894, 13.394)	1.211	(0.951, -0.187, -0.245)
		0.496	(0.053, -0.682, 0.729)
		0.328	(0.303, 0.707, 0.639)
C	(5.609, 4.060, 12.487)	0.686	(0.963, -0.288, 0.193)
		0.365	(0.259, 0.840, -0.477)
		0.234	(-0.073, 0.509, 0.858)
C	(5.405, 2.762, 11.791)	0.471	(0.985, -0.120, 0.121)
		0.323	(0.148, 0.954, -0.259)
		0.178	(-0.084, 0.273, 0.958)
N	(6.474, 4.920, 11.813)	0.546	(0.895, -0.435, -0.100)
		0.344	(0.420, 0.896, -0.147)
		0.213	(0.153, 0.089, 0.984)
O	(6.422, 2.120, 11.506)	0.507	(0.804, 0.582, -0.122)
		0.414	(-0.595, 0.787, -0.166)
		0.196	(-0.001, 0.206, 0.979)
O	(4.266, 2.444, 11.485)	0.632	(-0.162, 0.897, -0.411)
		0.465	(0.975, 0.209, 0.071)
		0.257	(-0.149, 0.390, 0.909)

HARMONIC NORMAL MODES

Harmonic zone-centre normal modes, calculated by the finite displacement method, are presented in Table S4. Molecular modes come in pairs for the 1/3 ML case, since there are two molecules per surface unit cell. The rms frequency splitting within each tabulated pair is just 19 cm^{-1}, but individual splittings can be as high as 57 cm^{-1}. Local-mode assignments are based upon (subjective) visual inspection of the phonon displacement patterns, and are presented in order of decreasing apparent contribution for each individual normal mode. Reconciliation with the RAIRS peaks reported by Barlow *et al* [*Surf. Sci.* **401**, 322-335 (1998)] is then based upon two considerations: proximity in frequency, and similarity of local-mode assignment.

For the peaks located experimentally at 2906 and 2860 cm^{-1}, correspondence to our calculated CH_2 stretch modes in the range 3048–3120 cm^{-1} is rather unambiguous. Similarly, the experimental peak at 1332 cm^{-1} seems fairly securely to correspond with our calculated normal modes at 1336 and 1347 cm^{-1}. On the other hand, the experimental peak at 1417 cm^{-1} could conceivably correspond either to our calculated modes at 1426 and 1431 cm^{-1} or to those at 1390 and 1400 cm^{-1}; the former pair lies closer in frequency on average, but the latter features a strongest contribution from symmetric CO_2 stretch vibrations, which fits better with the original deduction of Barlow *et al* [*ibid.*]. We therefore note both possible attributions in the table.

For our lowest-frequency normal modes, assignment to combinations of just a few local modes becomes much more difficult, so the correspondence with RAIRS is necessarily less certain. Nevertheless, the sparsity of the spectrum below 1100 cm^{-1} affords us some confidence in drawing a correspondence between the experimental peak at 969 cm^{-1} and our normal modes at 969 and 979 cm^{-1}. Furthermore, we also link the experimental peak at 945 cm^{-1} with our normal modes as 949 and 953 cm^{-1} despite their displacement patterns showing little evidence of the CH_2 rocking motion assigned previously to the experimental peak.

Unfortunately, we remain somewhat in the dark concerning the experimental peak at 1084 cm^{-1}, which was attributed by Barlow *et al* [*ibid.*] to a combination of NH_2 wag and CN stretch. Our harmonic calculations feature a pair of normal modes at 1174 and 1184 cm^{-1} lying on average 95 cm^{-1} too high in frequency, and another pair at 994 and 1043 cm^{-1} lying on average about 66 cm^{-1} too low. Neither pair shows clear evidence of NH_2 wag motion (although both show some CN stretch) but these are the only two options within at least 100 cm^{-1} of the correct frequency. We note that the pair of modes at 994 and 1043 cm^{-1} are very strongly split in frequency, despite appearing visually very similar in their displacement patterns, and speculate that they may be peculiarly sensitive to small variations in the local geometry. On this basis, and because they do lie on average closer than any other pair, we tentatively conclude that these are, in fact, the bands we seek.

With these correspondences in place, we compute the rms discrepancy between our harmonic normal-mode frequencies and those reported by Barlow *et al* [*ibid.*]. We include the two normal modes from each pair separately, rather than their average, and include both pairs that we have tentatively associated with the experimental peak at 1417 cm^{-1}. Altogether, this gives us sixteen terms in the sum, with the resulting rms discrepancy being 103 cm^{-1}. If we omit the CH_2 stretches from the sum, this reduces to an rms discrepancy of 31 cm^{-1}. Substituting percentage errors (relative to experimental frequencies) in place of absolute errors, we find an rms discrepancy of 4.2% over the sixteen modes for which we propose correspondences, reducing to an rms discrepancy of 2.8% if the CH_2 modes are omitted.

TABLE S4. Harmonic normal modes (in cm^{-1}) calculated by the finite displacement method.

1/6 ML	1/3 ML	Local Mode	RAIRS
3596	3550/3561	$\nu_a(NH_2)$	–
3514	3427/3438	$\nu_s(NH_2)$	–
3094	3109/3120	$\nu_a(CH_2)$	2906
3012	3048/3051	$\nu_s(CH_2)$	2860
1611	1632/1639	$\delta(NH_2)$, $\nu_a(CO_2)$	–
1552	1569/1574	$\nu_a(CO_2)$, $\delta(NH_2)$	–
1482	1496/1553	$\delta(CH_2)$, $\nu_a(CO_2)$	–
1394	1426/1431	$\rho(NH_2)$, $\nu_s(CO_2)$	1417
1331	1390/1400	$\nu_s(CO_2)$, $\omega(CH_2)$, $\rho(NH_2)$	1417
1259	1336/1347	$\omega(CH_2)$, $\nu_s(CO_2)$	1332
1156	1239/1245	$t(CH_2)$, $\omega(NH_2)$	–
1041	1174/1184	$t(CH_2)$, $\rho(NH_2)$, $\nu(CN)$, $\nu(CC)$	–
1020	994/1043	$\rho(CH_2)$, $\nu(CN)$, $\nu(CC)$	1084
962	969/979	$\nu(CN)$, $\nu(CC)$, $\nu_s(CO_2)$, $\omega(NH_2)$	969
926	949/953	$\nu(CN)$, $\omega(NH_2)$, $\omega(CH_2)$	945
734	789/792	$\delta(CO_2)$, $\delta(CCN)$	–
642	738/751	$\delta(CCN)$, $\rho(NH_2)$, $\omega(CO_2)$	–
550	651/653	$\nu(CC)$, $\omega(CO_2)$, $\delta(CO_2)$	–
542	528/530	$\rho(CO_2)$	–
